# Pepsin is a positive regulator of Ac-cathB-2 involved in the rat gut penetration of *Angiostrongylus cantonensis*

**DOI:** 10.1186/s13071-016-1568-4

**Published:** 2016-05-17

**Authors:** Ying Long, Binbin Cao, Yinan Wang, Damin Luo

**Affiliations:** School of Life Sciences, Xiamen University, Xiamen, Fujian 361102 China; Translational Medicine Center, Hunan Cancer Hospital, Hunan, 410006 China; State Key Laboratory of Cellular Stress Biology, Xiamen University, Xiamen, Fujian 361102 China; Medical College, Xiamen University, Xiamen, Fujian 361102 China

**Keywords:** *Angiostrongylus cantonensis*, Ac-cathB-2, Pepsin, Protease, Intestinal penetration

## Abstract

**Background:**

Angiostrongyliasis caused by the rat lungworm, *Angiostrongylus cantonensis* (*A. cantonensis*), has globally spread from the traditional epidemic areas. The small intestine is the site where the third-stage larvae (L3) of this worm entered the host body, and parasite proteases are involved in this process. Ac-cathB-2, a cathepsin B-like cysteine of *A. cantonensis,* was formerly isolated from the insoluble part of fragmentised *Escherichia coli* without activity. The unplanned low activity of prokaryotic expression proteins and difficulties in genetic modification hindered understanding the function of this protein.

**Methods:**

The recombinant Ac-cathB-2 was expressed and harvested from 293 T cells and the enzymatic property and the effects of processing on the activity of the recombinant protease were investigated in vitro. The expression of Ac-cathB-2 in response to external stimulation was assessed, and the function of this protease during host gut penetration was observed by using antiserum for inhibition.

**Results:**

Of the life-cycle stages studied, L3 expressed the highest level of Ac-cathB-2 gene and released the corresponding gene product from the body. The expression of this gene was rapidly upregulated after incubating L3 in small intestine homogenate of rat. Recombinant Ac-cathB-2 was harvested from 293 T cell culture medium. This protease was activated by pepsin-HCl and the enabled Ac-cathB-2 could subsequently digest laminin and fibronectin readily. Moreover, the small intestine isolated from rat was disrupted after incubating with the activated Ac-cathB-2, resulting in the detachment of epithelial cells. Antiserum treatment inhibited the hydrolytic ability of recombinant Ac-cathB-2 by 82.7 %, and also reduced the tissue penetration of activated L3 by 41.2 %. Additionally, pre-incubation of L3 with artificial gastric acid increased the number of penetrating larvae by 53.2 %, and this alteration could be partly blocked by antiserum treatment.

**Conclusion:**

We believe that Ac-cathB-2 from *A. cantonensis* might help the worm to penetrate the rat gut, because the protease was able to degrade the tissue components of host. Nevertheless, our results further indicated that host pepsin played a beneficial role in this process by cleaving Ac-cathB-2 for activation. Thus, Ac-cathB-2 may probably represent an important target for the control of *A. cantonensis* infection.

## Background

*Angiostrongylus cantonensis*, a parasitic nematode dwelling in the heart and pulmonary arteries of rats, is thought to be the primary etiologic agent of human eosinophilic meningitis or meningoencephalitis in accidental infections [1]. Recently, there is increasing awareness of a large number of cases all over the world, especially of several outbreaks in Asia and Pacific islands. Although mild and self-limiting for this neglected tropical disease, severe infections without proper treatments caused death [2–4]. Several clinical treatments using anthelmintic drugs have shown good results in China and Thailand [5–7]. However, the possibility of exacerbating neurological symptoms [8–10] stresses the need for more targeted drugs and more efficient treatment strategies.

The wall of the small intestine, a protective screen to prevent pathogens from invasion, is the site for the infective larvae (the third-stage larvae, L3) of *A. cantonensis* to enter the host body, thus molecules related to this process are thought to be particularly good candidate antigens for the development of new vaccines and drugs. There is considerable evidence that proteases are involved in many parasite-associated events, including ingestion, immune evasion, and tissue invasion and so on [11, 12]. Ac-cathB-2 (GenBank: ADQ57304.1), which has sequence identity to members of the cathepsin B-like cysteine protease family, is highly expressed in L3 of *A. cantonensis* as compared to the fifth-stage larvae (L5) and adults, and is predicted to be the component of excretory/secretory products (ESPs) [13]. However, *A. cantonensis* proteins have always been reported to be heterologously expressed in *E. coli* as inclusion bodies [14], inappropriate for research of the function downstream. Together with the difficulties in genetic manipulation, our understanding of the function of specific genes in *A. cantonensis*is is making slow progress.

In the present work, we attempted to express and purify Ac-cathB-2 from 293 T cell line to obtain the recombinant protease with biological activity for later experiments. Except for the enzymatic property and proteolytic activity of rAc-cathB-2, we still wondered whether the processing of this protease was essential for its biological activity. On this basis, antiserum was used to block the activity of the native Ac-cathB-2 to further investigate the function of this protease during the penetration of the intestine by larval *A. cantonensis*. The results are expected to produce a deeper understanding of the molecular events during the penetration of the wall of the small intestine by L3 and to establish a platform for functional studies of the specific secreted molecules related to this process.

## Methods

### Parasite collection

The *A. cantonensis* strain has been maintained in the laboratory through Sprague Dawley (SD) rats and snails (*Pomacea canaliculata*), and the protocol used for worm collection was analogous to that described previously [15]. The first-stage larvae (L1) of *A. cantonensis* were collected from infected rat faeces by using a 500 mesh sieve after 45 days post-infection (dpi). To remove the contamination by faecal bacteria, worms were washed five times with sterile water. Fresh rat faeces containing first-stage infective larvae were applied to the surface of lettuce fed to the snails for infection. Two or three weeks later, infected snails were cut into small pieces and strained through a 300 mesh sieve, and the second-stage larvae (L2) or L3 were collected under a dissecting microscope. Rats were infected by being fed L3-containing snails. The fourth- and fifth-stage larvae (L4 and L5) were dissected out from the brain of infected rats 20 and 28 dpi, respectively. Adult worms were obtained from the lung tissue of infected rats after mercy killing 45 dpi. After five washes with phosphate-buffered saline (PBS), female adults were cultured in RPMI 1640 medium (HyClone, Logan, USA) for one day, and eggs were harvested from the culture medium by centrifugation. The nematode specimens collected were preserved in a RNA store solution (TIANGEN, Beijing, China), a reagent for stabilisation and protection of the tissue RNA expression pattern, under -80 °C.

### Ethics statement

Mammals were managed and housed in the Xiamen University Laboratory Animal Centre. This study was performed in strict accordance with the Regulations for the Administration of Affairs Concerning Experimental Animals (as approved by the State Council of the People’s Republic of China). The protocol was approved by the Committee for the Care and Ethics of Laboratory Animals of Xiamen University (Permit Number: XMULAC2012-0122). All surgery was performed under sodium pentobarbital anaesthesia, and all efforts were made to minimise suffering.

### ESPs collection

Freshly isolated L3 were surface sterilised by incubating in 0.2 % NaClO for five minutes at room temperature followed by six washes in sterile PBS for removal of the residual NaClO. L5 and adults were collected separately from infected rats as described above in a sterile environment, washed three times with sterile PBS, and subsequently divided into four groups (female L5, male L5, female adult and male adult). All these worms were cultured separately in RPMI 1640 medium (HyClone, Logan, USA) supplemented with antibiotics in an atmosphere of 5 % CO_2_. The medium was collected every 24 h by centrifugation and refreshed for further culture. The in vitro culture lasted for no longer than 4 days. The medium was subsequently concentrated 50–100 fold using Amicon Ultra centrifugation tubes (Millipore, Waters, USA) with a molecular weight cut-off of 10 kDa after passing through a 0.22 μm filter (Millipore, Waters, USA); the resulting solutions were stored at -80 °C.

### Expression analysis of Ac-cathB-2 gene

Quantitative real-time PCR (qRT-PCR) was performed to measure the mRNA level of Ac-cathB-2 gene throughout the life-cycle stages of *A. cantonensis*. Since L3 move through the gut and penetrate the walls of the small intestine of the definitive host, there is a possibility that host varied tissues and gastric acid may have different effects on L3 or on the expression of Ac-cathB-2 gene.

The effects of tissue homogenates from different parts of the rat digestive tract on the expression of Ac-cathB-2 gene were tested by qRT-PCR. Accordingly, total RNAs of L3 induced by homogenates of oesophagus, stomach, small intestine and large intestine of rat for 2 h and 4 h separately in the sterilised Tyrode’s solution were extracted using Stool RNA Kit (OMEGA Biotek, Doraville, USA), and subsequently treated with DNase I before reverse transcription. In addition, L3 were also induced with Phosphate buffer saline (PBS), pepsin (10 mg/ml pepsin in 0.5 M sodium phosphate), pepsin-HCl (10 mg/ml pepsin in 0.5 M sodium phosphate, pH 3.0) and HCl (pH 3.0) for 0.5 h, respectively at the same time, and total RNA for all groups was extracted and treated for obtaining cDNA template as described above. All assays were done in triplicate and the expression of Ac-cathB-2 gene (qPrimer-cathB2-F: 5'-CGG AGC AGT TGA AGC AAT GAC-3', qPrimer-cathB2-R: 5'-ACA AGG TGG GTA TGG G-TA GGG-3') was presented as 2^-ΔΔCt^ using β-actin as an internal reference (qPrimer-actin-F: 5'-CCC AGA GCA GTC TTT CCT TCC A-3', qPrimer-actin-R: 5'-CCA TAG GGT ATT TCA GCG TTA G-3').

### Eukaryotic expression and purification of recombinant Ac-cathB-2 (rAc-cathB-2)

Excluding the native signal peptide in the open reading frame, the sequence of Ac-cathB-2 gene was amplified by high-fidelity PCR with specific primers (cPrimer-cathB2-F: 5'-CTC GAG GCA TCT TGG CAA AAT GCA AAG A-3', cPrimer-cathB2-R: 5'-GGA TCC TTT CGG TTC TCC GGC AAC G-3'; *Xho*I and *BamH*I restriction sites are underlined), and ligated into pEASY-T5 Zero cloning vector (TransGen Biotech, Beijing, China). The target sequence was then cut off and incorporated into the expression cassettes of pcDNA3.1- IgK vector by *Xho*I and *BamH*I to form the eukaryotic expression vector pcDNA3.1-IgK-cathB2. PEI-mediate transient transfection of 293 T with this expression vector was performed to produce the recombinant protein, and the protein could be easily purified because of being released into the cell culture supernatants. The cell culture medium was collected and the His-tagged rAc-cathB-2 could be purified using the immobilised metal affinity chromatography (Ni-NTA resin, GE Healthcare, Uppsala, Sweden). All washes and eluates were analysed by SDS-PAGE with Coomassie brilliant blue staining, and the end product was analysed by western blot using both antiserum against Ac-cathB-2 and commercial anti-His antibody for validation.

### Activation of rAc-cathB-2

One volume of rAc-cathB-2 solution (0.2 mg/ml in PBS, pH 7.0) combined with 0.5 volume of PBS, pepsin-HCl (10 mg/ml pepsin in 0.5 M sodium phosphate, pH 3.0) or HCl (pH 3.0), respectively, was incubated at 37 °C for 30 min in the presence of 5 mM DTT. The activation reaction was stopped by addition of pepstatin A (Sigma, MO, USA) to a final concentration of 1 mM.

The rAc-cathB-2 from all three groups listed above was incubated in the reaction buffer (100 mM Tris, 10 mM CaCl_2_, 5 mM DTT, pH5.5) at 37 °C to evaluate the alterations of proteolytic activity towards fluorescent substrate Z-Arg-Arg-7-amido-4-methylcoumarin hydrochloride (Z-RR-AMC, Sigma, MO, USA) according to the method described previously with slight modifications [16]. To rule out possible effects of pepsin to hydrolyze Z-RR-AMC, a control of pepsin-HCl without rAc-cathB-2 was incubated with Z-RR-AMC in the reaction buffer. The fluorescence of the released AMC was measured with excitation and emission wavelengths of 355 and 460 nm, respectively. The data are presented as relative activities of Ac-cathB-2 after background correction, and the average activity of the PBS-treated groups was taken as 1.

### Analysis of pH-dependence activity profile of rAc-cathB-2

The activity of rAc-cathB-2 was investigated by cleavage of Z-RR-AMC at 37 °C within a pH range of 3–10. The buffer was composed of 100 mM Tris, 10 mM CaCl_2_ and 5 mM DTT. After injection of activated rAc-cathB-2, the substrate was added at a final concentration of 50 μM to start the reaction. The fluorescence was measured, and the data were presented as describe above. The highest activity at the pH optimum was taken as 100 %.

### Hydrolysis activity of rAc-cathB-2

One microgram of activated rAc-cathB-2 was incubated for 6 h with various substrates (10 μg) found in connective tissues, including type I collagen, laminin and fibronectin (Sigma, MO, USA). Controls were performed using activated rAc-cathB-2 pre-treated with E64, an irreversible, potent, and highly selective cysteine protease inhibitor, especially for cathepsin B, and substrates adding only PBS served as blanks. After incubation, the samples were analysed with 5 % SDS-PAGE stained with Coomassie Brilliant Blue.

Further, the small intestine of rat was excised and transferred to sterilised Tyrode’s solution with or without 50 μg/ml activated rAc-cathB-2, and incubated for 2 h at room temperature. For microscopic observations, tissue pieces were fixed and embedded in paraffin and serial sections were stained with Mayer’s hematoxylin and eosin.

### Biological activity of rAc-cathB-2

PBS, antiserum against Ac-cathB-2 (positive serum), normal mice serum (negative serum) and 20 μM E64 were incubated with activated rAc-cathB-2 for 30 min prior to assessment of the degradation of Z-RR-AMC. Then, after background correction, the cleavage of the substrate was calculated as above.

To further investigate the relationship between Ac-cathB-2 and the host intestinal penetration by L3, the effects of positive serum and gastric acid on larval gut penetration was assessed. L3 were incubated inartificial gastric acid (pH 1.5, 1 % pepsin-1 % HCl) for 10 min for activation ahead of the assay. Equal numbers of normal L3 and activated L3 were pre-treated with PBS, positive serum, negative serum and E64 for 30 min. After incubation, L3 from different treatment groups were injected into the lumen of the excised segments of rat small intestine and kept in sterilised Tyrode’s solution for 3 h. The alterations in the behaviour of gut penetration by L3 were determined by counting the number of larvae remaining in the gut lumen.

### Statistics

All assays were performed in triplicate and the values were expressed as the mean ± standard deviation (SD). Significant differences between groups were analysed by one-way analysis of variance (ANOVA) followed by Duncan’s multiple comparison test with SPSS 13.0 (SPSS, Inc., Chicago, IL, USA), with a *P-*value < 0.05 considered statistically significant.

## Results

### L3 exhibited the highest expression of Ac-cathB-2 gene and released the protein into ESPs

**Fig. 1:**
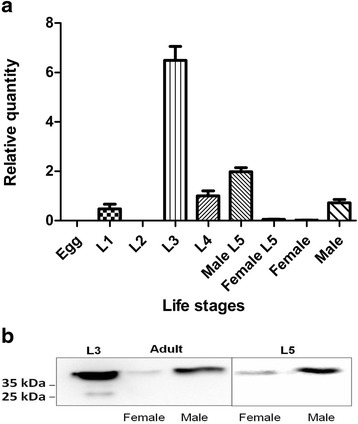
Differential expression of Ac-cathB-2 gene in the life cycle of *Angiostrongylus cantonensis*. **a** Quantitative real-time PCR (qRT-PCR) was used to measure the expression of Ac-cathB-2 gene throughout the life-cycle stages of *A. cantonensis,* with β-actin as an internal reference. The data are presented as relative quantities and the average amount of Ac-cathB-2 gene expression in male adults was taken as 1. **b** Equal amounts of ESPs from L3 (Lane 1), female adults (Lane 2), male adults (Lane 3), female L5 (Lane 4) and male L5 (Lane 5) were subjected to western blot assay. The native Ac-cathB-2 in these samples was visualised with the antiserum against Ac-cathB-2

The expression of Ac-cathB-2 gene was measured for each life-cycle stage of *A. cantonensis* by qRT-PCR. The results showed that the expression of Ac-cathB-2 gene differed significantly among different stages (ANOVA *F*_(8, 18)_ = 268.349, *P* < 0.0001). This gene was highly expressed in larval stages within the mammalian host; in particular, the highest level was detected in the infective stage L3. However, the expression of Ac-cathB-2 gene was much lower in L1 and male adults, whereas in the female L5 and female adults the expression has decreased to an insignificant level. The mRNA amounts of Ac-cathB-2 gene were not detected in the second-stage larvae (L2) and eggs (Fig. 1a).

Since Ac-cathB-2 was predicted to comprise a part of ESPs in our previous work [15], western blot assay was performed to confirm the relationship between ESPs of *A. cantonensis* and the native Ac-cathB-2; the result supported this prediction. Native Ac-cathB-2 was recognised in all five samples of ESPs from L3, male adults, female adults, male L5 and female L5 (Fig. 1b), indicating that the protease was a ESPs component. Additionally, as shown in Fig. 1b, L3 secreted remarkably higher amounts of Ac-cathB-2 than adults (Fig. 1b), which was consistent with the results of the qRT-PCR.

Except for the expected band, a slight band at a lower position was detected in the ESPs of L3 (lane 1 in Fig. 1b), indicating a protein cleavage for the activation might occur in the host-parasite interaction and accounting for the discrepancy between the predicted and observed molecular weights of Ac-cathB-2.

### The expression of Ac-cathB-2 gene was more sensitive to homogenate of rat small intestine

**Fig. 2:**
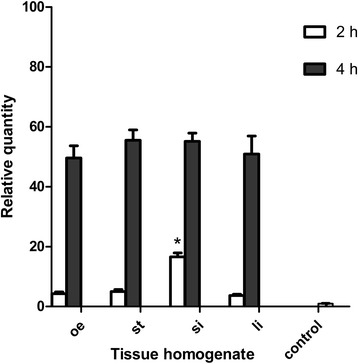
Differential expression of Ac-cathB-2 gene in L3 in response to host stimulation. The expression of Ac-cathB-2 gene in response to homogenates from oesophagus (oe), stomach (st), small intestine (si) and large intestine (li) detected by qRT-PCR at 2 h and 4 h. The expression of Ac-cathB-2 gene in L3 without any induction is used as control. The data are presented as relative quantities and the average amount of Ac-cathB-2 gene expression in the control was taken as 1. Asterisk indicates *P* < 0.05

Pre-incubations of L3 with homogenates from four different parts of the rat alimentary tract indicated upregulated Ac-cathB-2 gene expression (ANOVA *F*_(8, 18)_ = 22.683, *P* < 000.1), and no significant differences (ANOVA *F*_(3, 8)_ = 1.482, *P* = 0.29) were detected for the expression levels of L3 among the four treatments after 4 h incubation (Fig. 2). Interestingly, obviously higher amounts of Ac-cathB-2 mRNA (ANOVA *F*_(3, 8)_ = 169.645, *P* < 0.0001) were observed in L3 after 2 h induction with the homogenate of the small intestine in comparison with those of the oesophagus, stomach and large intestine, respectively (Fig. 2), suggesting that L3 might be more sensitive to small intestine.

### Expression and purification of rAc-cathB-2

**Fig. 3:**
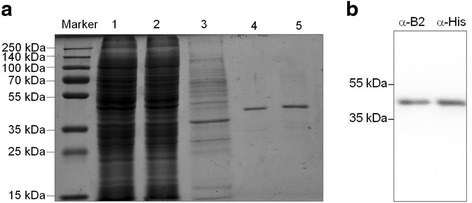
Purification and identification of recombinant Ac-cathB-2. a SDS-PAGE analysis of all fractions through Nickel resin for purification. Lanes: Marker, molecular weight marker; 1, concentrated culture supernatant in binding buffer; 2, column flow-through; 3, binding buffer wash; 4 and 5, successive eluate fractions. **b** Identification of the purified rAc-cathB-2 by Western blot with an antiserum against Ac-cathB-2 expressed in *E. coli* and an anti-His antibody

cDNA of Ac-cathB-2 was cloned and expressed in 293 T cells. The recombinant Ac-cathB-2 was purified at a yield of 2 mg/L culture medium after immobilised metal affinity chromatography (Ni-NTA resin, GE Healthcare, Uppsala, Sweden). The purified protein migrated with an apparent molecular mass around 40 kDa (Fig. 3a) and was recognised by western blotting with the anti-Ac-cathB-2 antibody as well as with a commercial anti-His antibody (Fig. 3b), implying that the rAc-cathB-2 was purified as expected.

### Gastric acid was involved in the processing of Ac-cathB-2

**Fig. 4:**
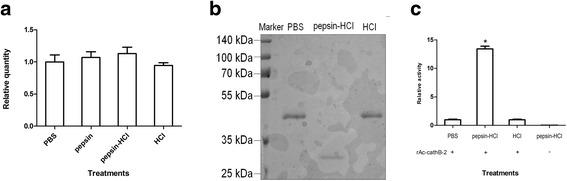
Gastric acid is involved in the processing of rAc-cathB-2. **a** The effects of pepsin, HCl and pepsin-HCl on the expression of Ac-cathB-2 gene were tested by qRT-PCR. No obvious effect on the mRNA amounts of Ac-cathB-2 gene was detected in comparison with the control (PBS). **b** Cleavage of rAc-cathB-2. Equal amounts of rAc-cathB-2 were incubated with PBS, pepsin-HCl and HCl for 0.5 h in the presence of 5 mM DTT. Lane 1, molecular weight markers; Lanes 2–4, different treatments as indicated; **c** Proteolytic activity of rAc-cathB-2 from all three groups above was assessed at 37 °C by degrading the fluorescent substrate Z-RR-AMC. A control of pepsin-HCl without rAc-cathB-2 was also incubated with Z-RR-AMC. The fluorescence of the released AMC was measured with excitation and emission wavelengths of 355 and 460 nm, respectively. The data are presented as relative activities of Ac-cathB-2 after background correction, and the average activity of the PBS-treated groups was taken as 1. Asterisk indicates *P* < 0.05

Some studies reported that pepsin-HCl was involved in the activation of *A. cantonensis* L3 [17]. Whether or not pepsin-HCl stimulated directly the expression of Ac-cathB-2 gene was tested by qRT-PCR. The results showed that pre-incubation of L3 with pepsin, HCl or pepsin-HCl for 0.5 h had no obvious effects on mRNA amounts of Ac-cathB-2 gene (ANOVA *F*_(3, 8)_ = 2.491, *P* = 0.13) in comparison with the control (PBS), suggesting that Ac-cathB-2 gene expression might not be in response to the gastric acid (Fig. 4a).

Subsequently, equal amounts of rAc-cathB-2 were incubated in PBS, pepsin-HCl and HCl for 0.5 h separately. In the presence of pepsin-HCl, the recombinant protein was digested into a smaller peptide with a molecular mass around 30 kDa as showed by PAGE analysis (Fig. 4b). This result was a little bit similar to the cleavage of human cathepsin B. At this time, we believed that pepsin-HCl might be involved in the processing of Ac-cathB-2 protease. Besides, significant effect of different incubations on the hydrolytic activity rAc-cathB-2 was observed (ANOVA *F*_(3, 8)_ = 122.475, *P* < 0.0001). Obviously, rAc-cathB-2 from pepsin-HCl treated group exhibited a more than 12-fold increase of hydrolytic ability against Z-RR-AMC at 37 °C in comparison with that from the PBS treated group (Fig. 4c). The result of the control show that pepsin-HCl was inactive to Z-RR-AMC. Thus, our results supported that pepsin-HCl could activate parasite enzyme in some way.

### Enzymatic assay of the activated rAc-cathB-2

**Fig. 5:**
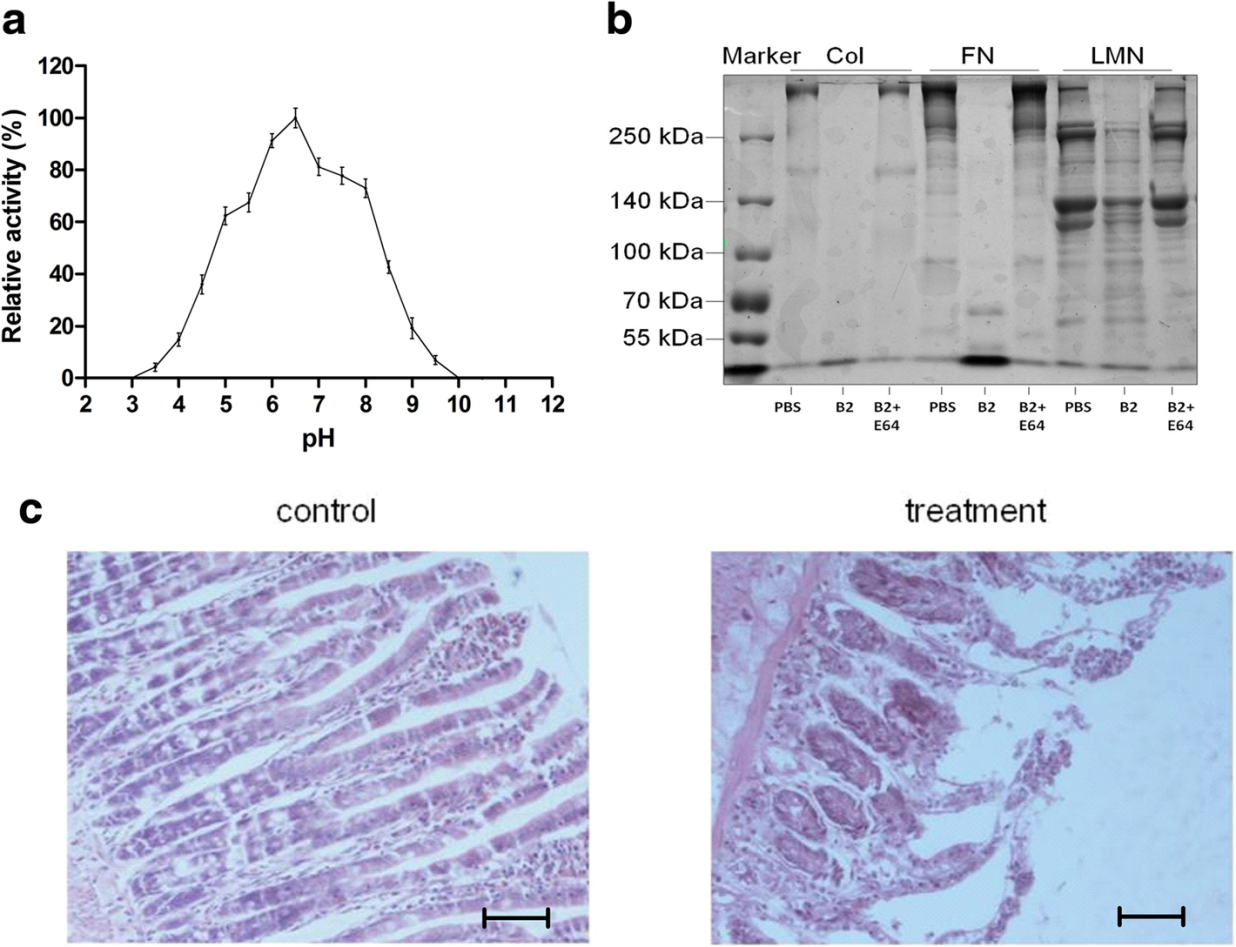
pH-dependence profile and hydrolysis activity of rAc-cathB-2. **a** pH-dependence profile of rAc-cathB-2 activity was measured with the specific substrate Z-RR-AMC. The data are presented as relative activities of Ac-cathB-2, where the highest activity at the pH optimum was taken as 100 %. **b** Digestive activity of rAc-cathB-2 on proteins of connective tissue components. After incubation for 6 h, all samples were fractioned in a 5 % gel followed by Coomassie brilliant blue staining. Lanes: 1, molecular weight markers; Type I collagen (Col), fibronectin (FN) and laminin (LMN) were incubated with PBS, Ac-cathB-2 or Ac-cathB-2 and E64 as indicated. **c** Influence of rAc-cathB-2 on isolated small intestine. After treating with rAc-cathB-2 for 2 h, the specimens were fixed and embedded in paraffin. Serial sections were stained with Mayer’s hematoxylin and eosin, and analysed under a light microscope. *Scale-bar*: 50 μm

To characterise the enzymatic property, the pH-dependence of activated rAc-cathB-2 was studied. The recombinant protease exhibited a roughly bell-shaped pH profile within the pH range of 3.5–9.5 with an optimum around pH 6.5 and showed much lower activity under strong acid and strong alkaline environment (Fig. 5a). As a consequence, this protease was thought to be active in the physiological conditions of the lumen of the small intestine, within the range of pH of approximately 6.0–7.4.

Further, rAc-cathB-2 was incubated with the proteins found in the connective tissue at neutral pH to simulate the intraluminal pH of the small intestine, and the results showed that the recombinant protease was catalytically active towards the substrates being tested and its proteolytic activity could be completely inhibited by E64. After 6 h at 37 °C, rAc-cathB-2 completely degraded fibronectin and laminin, but only partially digested type I collagen (Fig. 5b).

Furthermore, clear signs of tissue disarrangements were observed under the light microscope after treating the excised rat small intestine with rAc-cathB-2 for 2 h. The treatment severely damaged the gut mucosa with evident alterations, such as shrinking of the whole intestinal wall, detachment of epithelial cells from the basal lamina, even detachment of a fraction of the cells into the lumen (Fig. 5c).

### Ac-cathB-2 activated by artificial gastric acid was positive to gut penetration by L3

**Fig. 6:**
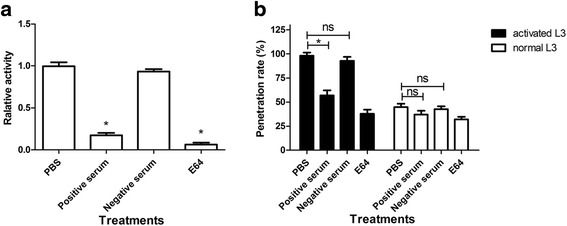
Biological activity of Ac-cathB-2 in the host intestinal penetration of L3. **a** Digestion of Z-RR-AMC by rAc-cathB-2 and inhibition of proteolytic digestion by antiserum to Ac-cathB-2 was assessed. The activated rAc-cathB-2 was incubated with PBS, antiserum against Ac-cathB-2 (positive serum), normal mice serum (negative serum) and 20 μM E64. **b** Effects of positive serum and gastric acid on larval gut penetration were assessed. Two hundred of activated L3 and normal L3 were respectively incubated with the positive serum, the negative serum, PBS and E64 for 30 min. After incubation, the ability of gut penetration for L3 was determined by counting the number of larvae remaining in the gut lumen, and the data were presented as the penetration rate. Asterisk indicates *P* < 0.05; ns, not significant

The inhibition of proteolytic activity of rAc-cathB-2 was assessed in vitro to investigate the effects of Ac-cathB-2 on the penetration of rat intestine by L3. As shown in Fig. 6a, the effect of four treatments differed significantly (ANOVA *F*_(3, 8)_ = 218.732, *P* < 0.0001). In comparison with PBS-treated group, pre-treatment of rAc-cathB-2 with the positive antiserum reduced the hydrolysis of the fluorescent substrate by 82.7 % whereas just an insignificant reduction was detected in the group immunised with the normal mice serum; and a 97.3 % decrease of the enzymatic activity was achieved in the presence of E64.

Results from the tests designed for exploring the involvement of the gastric acid and Ac-cathB-2 in the gut penetration by L3 showed that different treatments exhibited significant effects on L3 (ANOVA *F*_(7, 16)_ = 137.135, *P* < 0.0001). As for the activated L3, the penetration ability of L3 differed significantly between the four treatments (ANOVA *F*_(3, 8)_ = 133.202, *P* < 0.0001). The antiserum incubation prevented 41.2 % of activated L3 from penetrating the intestinal wall in vitro, whereas negative mice serum resulted in just insignificant reduction in larval migration (Fig. 6b). Pre-incubation with E64 also reduced the number of activated L3 that successfully penetrated the intestinal wall by 59.2 %. These results indicate that the inhibition of the enzymatic activity of Ac-cathB-2 suppressed the gut penetration ability of L3. As in the normal L3 groups, different treatments also had significant effects on penetration abilities of L3 (ANOVA *F*_(3, 8)_ = 9.582, *P* = 0.005). However, there was no remarkable difference between the antiserum-treated group and the PBS-treated group. This was probably due to the fact that few Ac-cathB-2 was activated in normal L3. In addition, in comparison with the normal L3, 53.2 % more L3 entered into the intestinal tissue in the pepsin-HCl-treated group, and this effect of pepsin-HCl treatment was partially blocked by inhibition of the enzymatic activity of Ac-cathB-2 with antiserum, further suggesting that host gastric acid was a positive regulator of larval penetration ability through activating Ac-cathB-2.

## Discussion

The present study indicates that Ac-cathB-2 secreted by *A. cantonensis* was likely to help L3 in penetrating the wall of the rat small intestine. The rAc-cathB-2 was shown to be activated by artificial gastric acid and was able to degrade laminin, fibronectin, and type I collagen. However, it is still necessary to confirm whether Ac-cathB-2 must undergo the activation of host gastric acid in the process of invasion. Moreover, antiserum against Ac-cathB-2 greatly reduced larval penetration of the intestinal wall in vitro, strongly suggesting the involvement of Ac-cathB-2 in this process.

Sequence analysis of Ac-cathB-2 proves that it belongs to cathepsin B family [13], which is a type of ubiquitously expressed cysteine proteases. Unlike commonly in the lysosome of mammalian cells, cathepsin Bs of many parasites are secreted from the cell and involved in the pathogenesis, immune evasion, migration, nutrition and so on [18, 19]. To investigate the stage specificity of Ac-cathB-2 gene throughout the life-cycle, qRT- PCR analysis of this gene in all life-cycle stages was performed, and the result showed that L3 possess the highest expression of Ac-cathB-2 gene. Chang et al*.* [17] reported that the Ac-cathB-2 mRNA was one of the most abundantly expressed transcripts in L3. In addition, our results imply that this protease probably plays a key role in the L3 stage. In our previous study, the location of Ac-cathB-2 was labelled in the digestive tract, the body wall and the excretory tube of L3 [15]. Western blot assay confirmed that Ac-cathB-2 was released by the worm into the environment as a part of ESPs, and this result was in consistent with the prediction of our previous study [15]. When the host ingests food containing L3, the larvae enter the host body from the digestive tract, and proteases in the ESPs are thought to be particularly central to tissue penetration. Thus we checked the relationship between the tissues of the host digestive tract and Ac-cathB-2 gene expression in L3. Although the expression of this gene showed an ascending tendency in response to all tested homogenates and no differences were detected after enough incubation, L3 sensed the small intestine homogenate more rapidly in comparison with the others. Therefore, this result was consistent with the fact that L3 enter the host body through the wall of the small intestine.

Heterologous expression of the encoding genes was helpful for biochemical characterisation and function explorations of parasite proteins in vitro. As reported in many studies, genes of *A. cantonensis* were expressed in *E. coli* for high yield and easy operation [20]. Although several gene products from the prokaryotic expression system were reported to be active for planning analysis, the physical and chemical property studies using recombinant proteins more close to nature may be more reasonable. In consideration the deficiency of modification after translation in the prokaryotic expression system, we harvested rAc-cathB-2 with biological activity from the mammalian cell line 293 T as a zymogen form. Pepsin-HCl was found to cleave the recombinant protein into *c*.30 kDa fragment, analogous to the cleavage of human cathepsin B [21]. No alteration was detected in groups of rAc-cathB-2 treated with PBS or HCl, implying that rAc-cathB-2 could not be activated by autocatalysis. Together with the increase of hydrolysis activity of Z-RR-AMC after pepsin-HCL treatment, our observation suggest that Ac-cathB-2 rely on *trans*-processing for activation, in accordance with the report that aspartic protease participated in the processing of cathepsin Bs [22, 23]. With the activated rAc-cathB-2, high activity of this protease was observed between pH of 5.0–8.5, similar to the values reported for the human tissue homogenate and purified enzyme [24, 25]. Moreover, the high activity of this protease at pH of *c*.8.0, differing from some other cathepsin Bs of *A. cantonensis* [26, 27], made it possible to work in a weak alkaline condition.

In parasitic worms, functional observations of cathepsin Bs were always focused on the assistance of L3 in the immune evasion and nutrition [27–29]. Although McGonigle et al. [30] reported that *Fasciola hepatica* cathepsin B has proven involvement in tissue invasion by RNAi, this knockdown method was refractory in *A. cantonensis*. Transcription profile assays, immunolocalisation and in vitro assays were used to define the function of genes in this species [13, 15, 17, 26], but they could not provide a direct proof. In mammalian studies, antibody was frequently applied to block the interaction between ligand and receptor with high specificity based on antigen-antibody reaction. In consideration the nonspecific effect of commercial inhibitors, we investigated the role of Ac-cathB-2 in gut penetration by inhibition of its enzymatic activity with antiserum. By means of this approach, we blocked the catalytic activity of Ac-cathB-2 at the rate of 82.7 % in vitro and reduced the number of activated L3 that penetrated into the isolated gut tissue by 41.2 %, indicating the function of this protease in aiding larval penetration. Lee et al. [31] reported that only serine protease and metalloprotease in ESPs of L3 are associated with larval penetration of the intestinal wall, which is different from our results. It seems that pepsin-HCl activation or lack of it could help interpret the divergence. Antiserum had a similar inhibition effect towards the proteolytic activity of inactivated Ac-cathB-2 as it did for the activated Ac-cathB-2 (data not shown). However, as shown in this study, it exhibited an insignificant effect on the larval penetration ability of normal L3, which was quite different from the condition in activated L3. Several studies pointed out that pepsin-HCl treatment not only recovered the L3 [32] but also activated the larvae [17]. Our results suggest that gastric acid did not affect the expression of Ac-cathB-2 gene, but positively regulated its activity in a post-translational cleavage mode. Pepsin-HCl treatment increased the number of L3 penetrating into the isolated gut, and the inhibition of this positive effect by incubating L3 with the positive serum confirmed that gastric acid positively affected intestinal penetration, and the impact was partially attributed to the activation of Ac-cathB-2. Based on the results above, we deduced that gastric acid and Ac-cathB-2 is probably involved in the host gut penetration of L3. When L3 reach the host stomach, Ac-cathB-2 is activated by gastric acid, but this activated protease is not active due to the strong acid conditions. As the larvae reach into the lumen of the small intestine, the hydrolytic activity of the activated Ac-cathB-2 is strengthened by the weak alkaline environment, facilitating the tissue invasion by L3.

## Conclusion

The activated Ac-cathB-2 of L3 is involved in intestinal penetration and migration through the connective tissues, and the weak alkaline environment of the host small intestine appears to facilitate the hydrolytic activity of Ac-cathB-2.

## Abbreviations

L1: first-stage larvae
L2: second-stage larvae
L3: third-stage larvae
L5: fifth-stage larvae
rAc-cathB-2: recombinant Ac-cathB-2
ESPs: excretory/secretory products
qRT-PCR: quantitative real-time PCR
Z-RR-AMC: Z-Arg-Arg-7-amido-4-methylcoumarin hydrochloride
SD: standard deviation
ANOVA: Analysis of Variance
PBS: Phosphate buffer saline
